# Supplementary figures and images for: Culturing-Enriched Metabarcoding Analysis of the Oryctes rhinoceros Gut Microbiome
Source: Insects. 2020 Nov 11;11(11):782. doi: 10.3390/insects11110782 (PMC7696363; doi:10.3390/insects11110782)

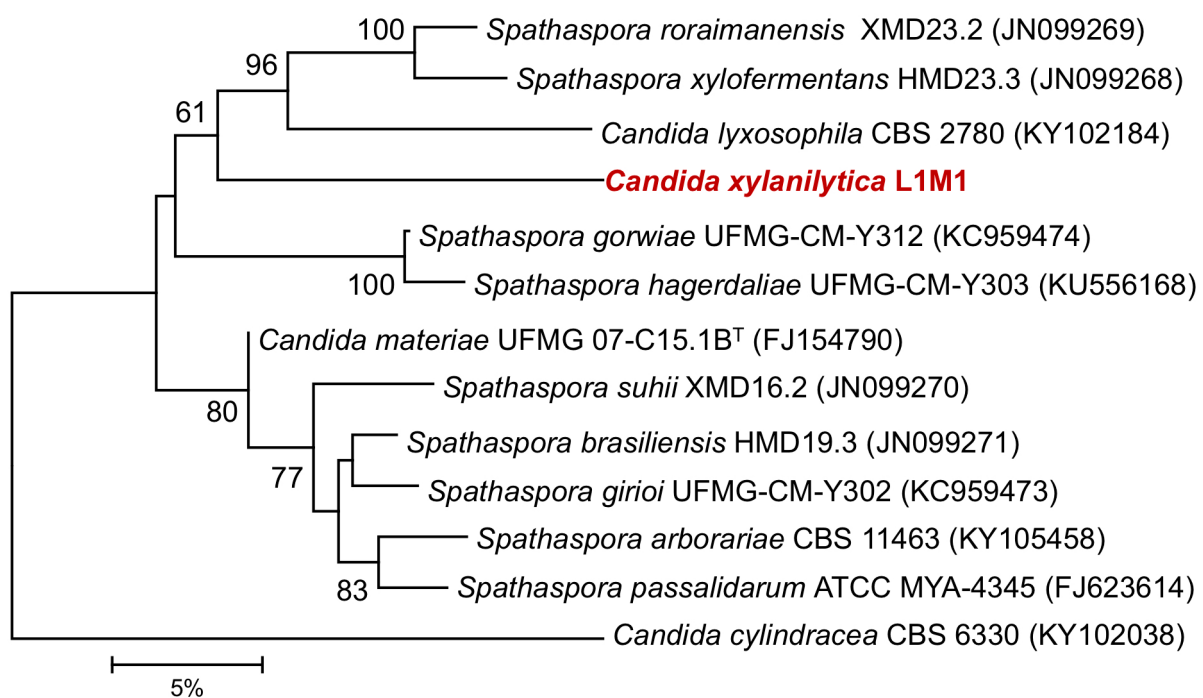

Supplement: Supplementary file 1 [file insects-11-00782-s001.zip › FigureS2.pdf]

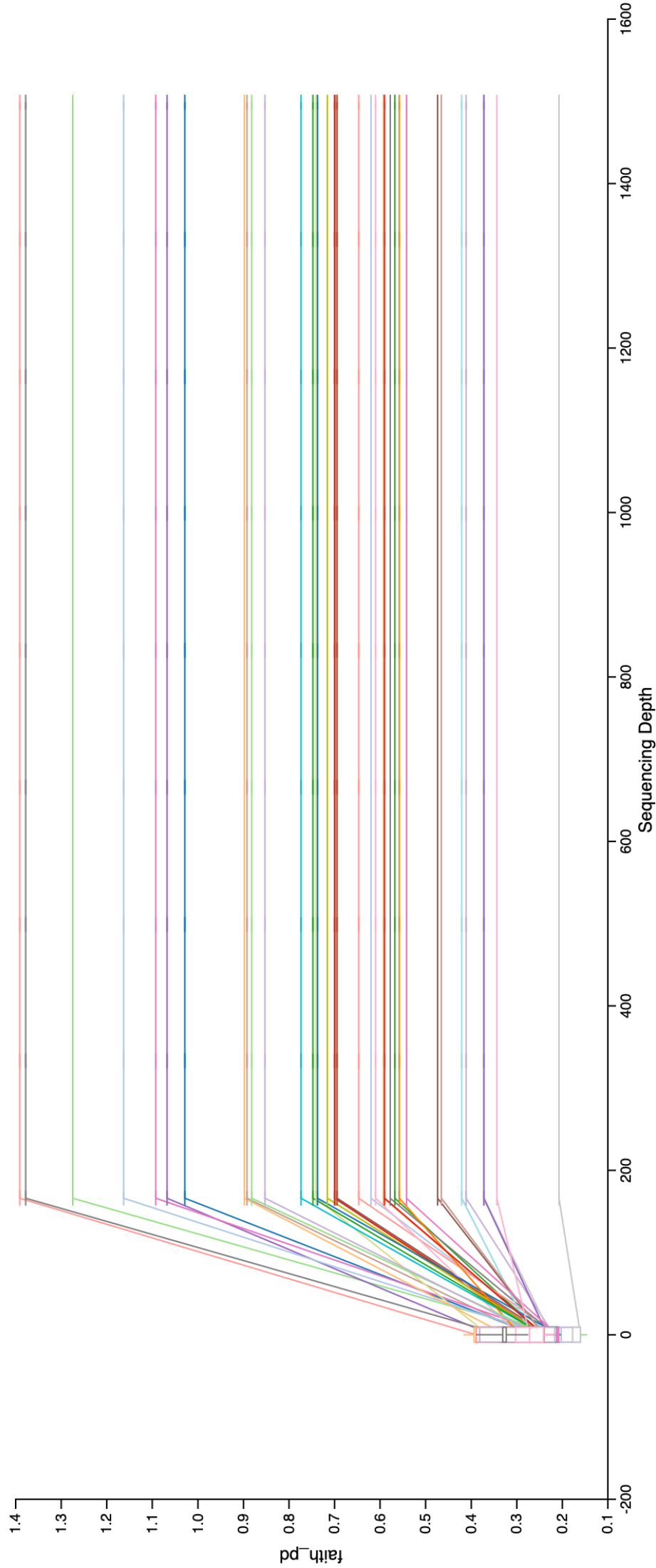

Supplement: Supplementary file 1 [file insects-11-00782-s001.zip › FigureS3.pdf]

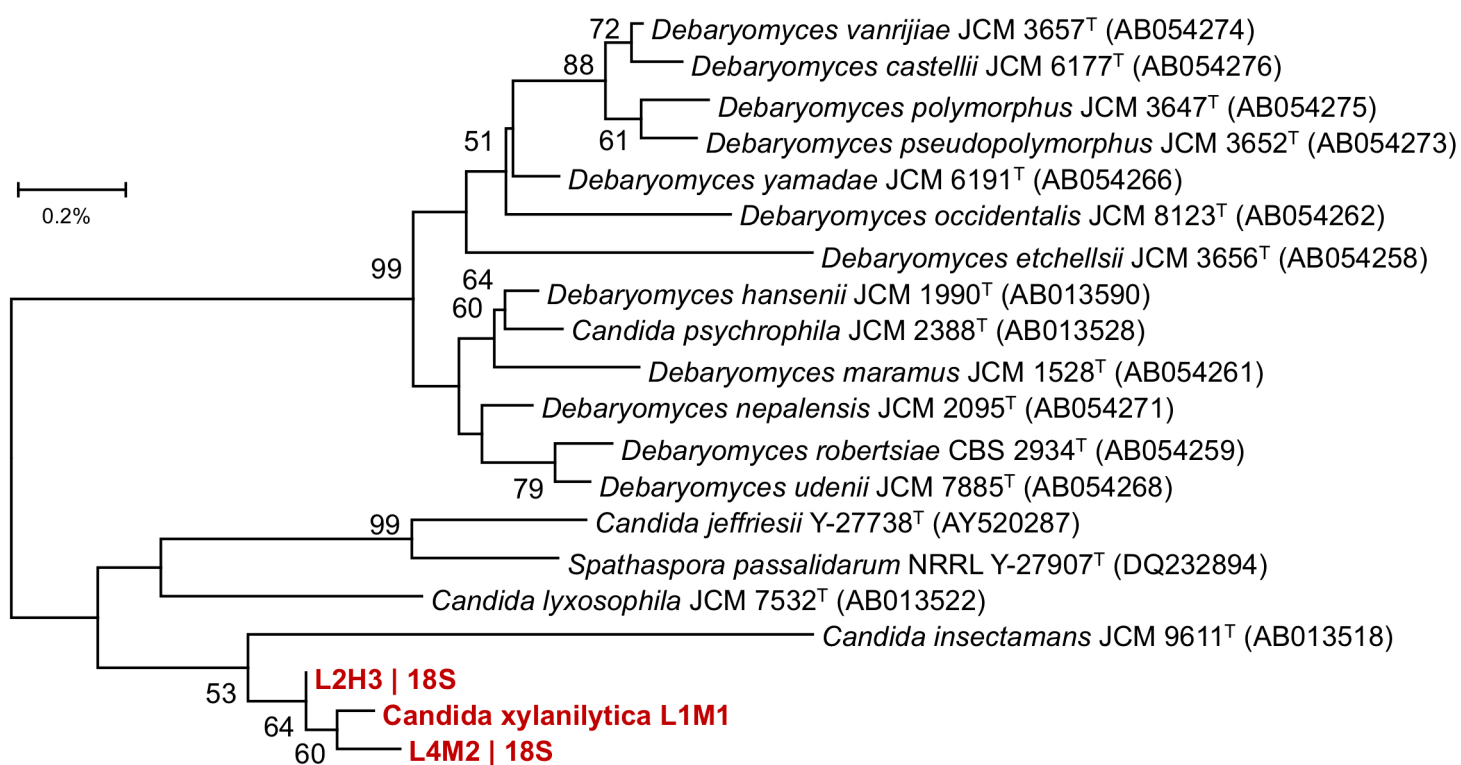

Supplement: Supplementary file 1 [file insects-11-00782-s001.zip › FigureS1.pdf]
